# 2-Amino-4-methyl­pyridinium 6-carb­oxy­pyridine-2-carboxyl­ate sesquihydrate

**DOI:** 10.1107/S1600536810046866

**Published:** 2010-11-20

**Authors:** Mahboubeh A. Sharif, Masoumeh Tabatabaee, Mahnaz Adinehloo, Hossein Aghabozorg

**Affiliations:** a Department of Chemistry, Islamic Azad University, Qom Branch, Qom, Iran; b Department of Chemistry, Islamic Azad University, Yazd Branch, Yazd, Iran; c Department of Chemistry, Islamic Azad University, North Tehran Branch, Tehran, Iran

## Abstract

In the title compound, C_6_H_9_N_2_^+^·C_7_H_4_NO_4_^−^·1.5H_2_O, extensive O—H⋯O, O—H⋯N, N—H⋯O and C—H⋯O hydrogen bonds, as well as ion pairing, π–π stacking inter­actions [centroid–centroid distances = 3.4690 (8) and 3.6932 (8) Å between aromatic rings] occur in the crystal. There are hydrogen-bonding inter­actions between water mol­ecules, which result in cyclic tetra­meric water clusters. One of the water O molecules has half occupancy. In the anion molecules, the –CO_2_ and –CO_2_H groups make torsion angles of 1.73 (18) and −12.14 (18)° with respect to the ring.

## Related literature

For background to hydrogen bonding involving water, see: Long *et al.* (2004 ▶); Atwood *et al.*, 2001 ▶); Miyake & Aida (2003 ▶). For related structures, see: Aghabozorg *et al.* (2008 ▶); Tabatabaee *et al.* (2009 ▶). 
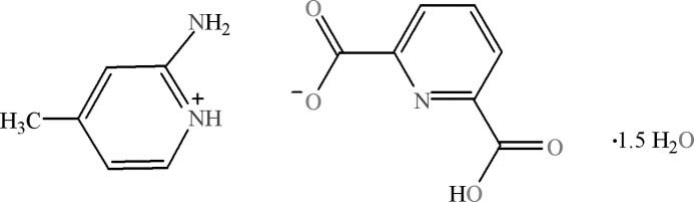


## Experimental

### 

#### Crystal data

C_6_H_9_N_2_^+^·C_7_H_4_NO_4_^−^·1.5H_2_O
*M* *_r_* = 302.29
Monoclinic, 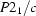

*a* = 9.2373 (6) Å
*b* = 7.1972 (5) Å
*c* = 21.6495 (14) Å
β = 93.951 (1)°
*V* = 1435.90 (17) Å^3^
*Z* = 4
Mo *K*α radiation
μ = 0.11 mm^−1^
*T* = 120 K
0.20 × 0.20 × 0.10 mm

#### Data collection

Bruker SMART 1000 CCD area detector diffractometer
Absorption correction: multi-scan (*SADABS*; Bruker, 1998 ▶) *T*_min_ = 0.980, *T*_max_ = 0.995
15297 measured reflections
3801 independent reflections
3077 reflections with *I* > 2.0σ(*I*)
*R*_int_ = 0.026

#### Refinement

*R*[*F* ^2^ > 2σ(*F* ^2^)] = 0.047
*wR*(*F* ^2^) = 0.102
*S* = 0.99
3801 reflections
216 parameters
H atoms treated by a mixture of independent and constrained refinement
Δρ_max_ = 0.36 e Å^−3^
Δρ_min_ = −0.25 e Å^−3^

### 

Data collection: *SMART* (Bruker, 1998 ▶); cell refinement: *SAINT-Plus* (Bruker, 1998 ▶); data reduction: *SAINT-Plus*; program(s) used to solve structure: *SHELXS97* (Sheldrick, 2008 ▶); program(s) used to refine structure: *SHELXL97* (Sheldrick, 2008 ▶); molecular graphics: *SHELXTL* (Sheldrick, 2008 ▶); software used to prepare material for publication: *SHELXTL* (Sheldrick, 2008 ▶).

## Supplementary Material

Crystal structure: contains datablocks I. DOI: 10.1107/S1600536810046866/pv2352sup1.cif

Structure factors: contains datablocks I. DOI: 10.1107/S1600536810046866/pv2352Isup2.hkl

Additional supplementary materials:  crystallographic information; 3D view; checkCIF report

## Figures and Tables

**Table 1:** Hydrogen-bond geometry (Å, °)

*D*—H⋯*A*	*D*—H	H⋯*A*	*D*⋯*A*	*D*—H⋯*A*
O1*W*—H1*WA*⋯O4^i^	0.85	2.01	2.846 (2)	169
O1*W*—H1*WB*⋯O3	0.85	1.97	2.813 (2)	169
O2*W*—H2*WB*⋯O1*W*^ii^	0.85	2.11	2.944 (2)	167
O2*W*—H2*WA*⋯O1*W*	0.85	2.07	2.919 (2)	175
O1—H1*O*⋯O1*W*^iii^	0.93 (3)	1.78 (3)	2.661 (2)	155 (2)
N2—H2*N*⋯O4	0.96 (2)	1.74 (2)	2.700 (2)	174 (2)
N3—H3*NB*⋯O3	0.98 (2)	1.86 (2)	2.829 (2)	172 (2)
N3—H3*NA*⋯O2^iv^	0.90 (2)	2.09 (2)	2.955 (2)	160 (2)
C2—H2*A*⋯O2^v^	0.95	2.47	3.158 (2)	129
C9—H9*A*⋯O1^iv^	0.95	2.56	3.399 (2)	147
C11—H11*A*⋯O2*W*^vi^	0.95	2.55	3.405 (2)	149

Symmetry codes: (i) 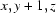; (ii) 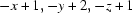; (iii) 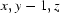; (iv) 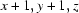; (v) 
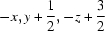
; (vi) 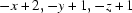.

**Fig. 1.**
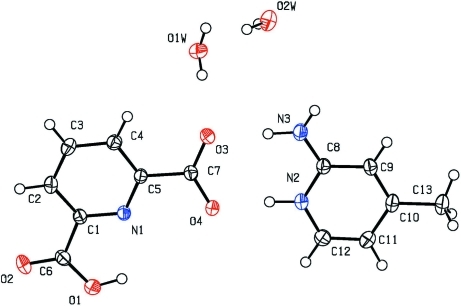
The asymmetric unit of (I), showing the atom numbering scheme. Displacement ellipsoids are drawn at the 50% probability level.

**Fig. 2.**
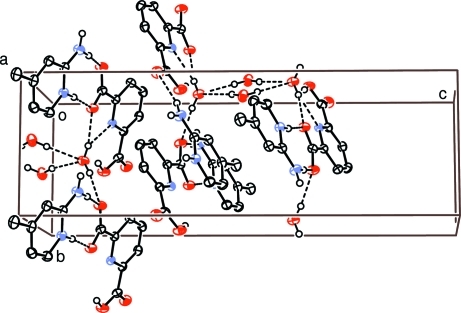
A packing diagram of (I) showing hydrogen bonds as dashed lines. Hydrogen atoms not involved in H-bonds have been excluded for clarity.

**Fig. 3.**
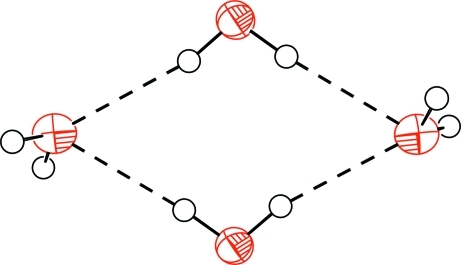
Tetrameric water cluster formed by H-bonds between water molecules in the title compound.

**Fig. 4.**
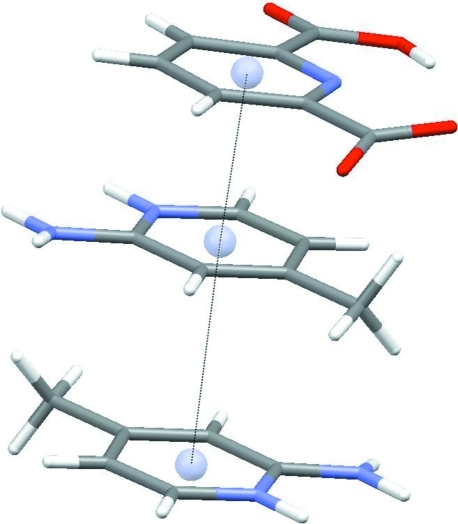
A view of the π-π stacking interaction between aromatic rings of the pyridine-2-carboxylate-6-carbonic acid and 2-amino-4-picolinium.

**Fig. 5.**
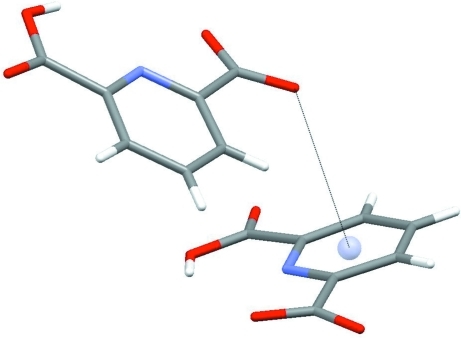
A view of the C═-O···π interaction between C7═O3 group and the centroid of the N1/C1-C5 aromatic ring of the anion, (pydcH)-.

## supplementary crystallographic information

### Comment

The presence of water is important in establishing H-bonded contributions to the total lattice energy, and is significant in establishing the stability of the hydrated crystal structures (Long *et al.*, 2004). Several water clusters found in organic or metallo-organic crystal hosts have been structurally characterized (Atwood *et al.*, 2001). A detailed understanding of the numerous possible structures and stability of isolated water clusters in diverse surroundings can help us understand the nature of water-water interactions in bulk water or ice. In this paper, we report the synthesis and crystal structure of the title proton transfer system, (I), derived from pyridine-2,6-dicarboxylic acid (pydcH_2_) and 2-amino-4-methylpyridine (2a4mp).

In the title compound, the asymmetric unit contains a cation, (2a4mpH)^2+^, an anion, (pydcH)^-^ and 1.5 water molecules (Fig. 1). The bond distances and bond angles in the title compound are in agreement with the corresponding distances and angles reported in some related crystal structures (Aghabozorg *et al.*, (2008); Tabatabaee *et al.*, (2009). In the crystal structure, the cations and the anions are linked by hydrogen bonds (Tab. 1 and Fig. 2). In the structure, water molecules form cyclic tetrameric water clusters (Tab. 1 and Fig. 3) in the most stable pattern (Miyake & Aida, 2003). The clusters play a bridging role (Fig. 2), linking the adjacent cations and anions *via* hydrogen bonds and contributing to the formation of an extensive supramolecular structure.

Moreover, π–π stacking interactions with distances between ring centroids = 3.4690 (8) Å and 3.6931 (8)Å, (Fig. 4) together with C7═O3···π involving aromatic ring of (pydcH)^-^ (Fig. 5) seem to be effective in stabilizing the crystal structure.

### Experimental

An aqueous solution of 2a4mp (324 mg, 3 mmol) in water (10 ml) was added to a stirring solution of pydcH_2_ (501 mg, 3 mmol) in water (10 ml). The reaction mixture was stirred at 298 K for 2h. Colorless crystals of the title compound were obtained by slow concentration of the solution at room temperature.

### Refinement

One of the water molecules (O2W) has 0.5 occupancy factor. The hydrogen atoms of OH, NH and NH_2_ groups and water molecules were found in difference Fourier synthesis. The H-atoms of OH, NH and NH_2_ groups were refined in isotropic approximation. The rest of the H-atoms were refined in riding model with C–H = 0.95 and 0.98 Å for aryl and methyl H-atoms and O–H = 0.85 Å for water molecules. The *U*_iso_(H) parameters were 1.2 *U*_eq_(C_aryl_/O) and 1.5 *U*_eq_(C_methyl_).

### Crystal data

**Table:**

C_6_H_9_N_2_^+^·C_7_H_4_NO_4_^−^·1.5H_2_O	*F*(000) = 636
*M**_r_* = 302.29	*D*_x_ = 1.398 Mg m^−^^3^
Monoclinic, *P*2_1_/*c*	Mo *K*α radiation, λ = 0.71073 Å
Hall symbol: -P 2ybc	Cell parameters from 1125 reflections
*a* = 9.2373 (6) Å	θ = 2–25°
*b* = 7.1972 (5) Å	µ = 0.11 mm^−^^1^
*c* = 21.6495 (14) Å	*T* = 120 K
β = 93.951 (1)°	Rhombic, colorless
*V* = 1435.90 (17) Å^3^	0.20 × 0.20 × 0.10 mm
*Z* = 4	

### Data collection

**Table:**

Bruker SMART 1000 CCD area detector diffractometer	3801 independent reflections
Radiation source: fine-focus sealed tube	3077 reflections with *I* > 2.0σ(*I*)
graphite	*R*_int_ = 0.026
φ and ω scans	θ_max_ = 29.0°, θ_min_ = 1.9°
Absorption correction: multi-scan (*SADABS*; Bruker, 1998)	*h* = −12→12
*T*_min_ = 0.980, *T*_max_ = 0.995	*k* = −9→9
15297 measured reflections	*l* = −29→29

### Refinement

**Table:**

Refinement on *F*^2^	Primary atom site location: structure-invariant direct methods
Least-squares matrix: full	Secondary atom site location: difference Fourier map
*R*[*F*^2^ > 2σ(*F*^2^)] = 0.047	Hydrogen site location: mixed
*wR*(*F*^2^) = 0.102	H atoms treated by a mixture of independent and constrained refinement
*S* = 0.99	*w* = 1/[σ^2^(*F*_o_^2^) + (0.0244*P*)^2^ + 1.3516*P*] where *P* = (*F*_o_^2^ + 2*F*_c_^2^)/3
3801 reflections	(Δ/σ)_max_ = 0.001
216 parameters	Δρ_max_ = 0.36 e Å^−^^3^
0 restraints	Δρ_min_ = −0.25 e Å^−^^3^

### Special details

**Table:**

Geometry. All e.s.d.'s (except the e.s.d. in the dihedral angle between two l.s. planes) are estimated using the full covariance matrix. The cell e.s.d.'s are taken into account individually in the estimation of e.s.d.'s in distances, angles and torsion angles; correlations between e.s.d.'s in cell parameters are only used when they are defined by crystal symmetry. An approximate (isotropic) treatment of cell e.s.d.'s is used for estimating e.s.d.'s involving l.s. planes.
Refinement. Refinement of *F*^2^ against ALL reflections. The weighted *R*-factor *wR* and goodness of fit *S* are based on *F*^2^, conventional *R*-factors *R* are based on *F*, with *F* set to zero for negative *F*^2^. The threshold expression of *F*^2^ > σ(*F*^2^) is used only for calculating *R*-factors(gt) *etc*. and is not relevant to the choice of reflections for refinement. *R*-factors based on *F*^2^ are statistically about twice as large as those based on *F*, and *R*- factors based on ALL data will be even larger.

### Fractional atomic coordinates and isotropic or equivalent isotropic displacement parameters (Å^2^)

**Table:**

	*x*	*y*	*z*	*U*_iso_*/*U*_eq_	Occ. (<1)
O1	0.26470 (12)	−0.06479 (14)	0.64545 (5)	0.0309 (2)	
H1O	0.356 (3)	−0.019 (3)	0.6373 (11)	0.066 (7)*	
O2	0.06351 (11)	0.00989 (15)	0.68870 (5)	0.0325 (2)	
O3	0.64297 (10)	0.62489 (14)	0.66239 (5)	0.0288 (2)	
O4	0.65846 (10)	0.32081 (14)	0.64256 (5)	0.0264 (2)	
O1W	0.53707 (11)	0.96510 (15)	0.61389 (5)	0.0305 (2)	
H1WA	0.5713	1.0680	0.6275	0.037*	
H1WB	0.5756	0.8707	0.6320	0.037*	
O2W	0.6530 (2)	1.0929 (3)	0.49926 (10)	0.0310 (5)	0.50
H2WB	0.5892	1.0666	0.4704	0.037*	0.50
H2WA	0.6142	1.0552	0.5315	0.037*	0.50
N1	0.38249 (11)	0.26733 (15)	0.67222 (5)	0.0192 (2)	
N2	0.91543 (12)	0.37469 (16)	0.59293 (5)	0.0223 (2)	
H2N	0.822 (2)	0.364 (3)	0.6099 (9)	0.043 (5)*	
N3	0.93113 (13)	0.67564 (18)	0.62910 (6)	0.0270 (3)	
H3NB	0.835 (2)	0.658 (3)	0.6447 (8)	0.037 (5)*	
H3NA	0.986 (2)	0.776 (3)	0.6395 (9)	0.044 (5)*	
C1	0.25047 (13)	0.23786 (18)	0.69207 (6)	0.0199 (2)	
C2	0.17514 (14)	0.3666 (2)	0.72513 (6)	0.0232 (3)	
H2A	0.0818	0.3388	0.7386	0.028*	
C3	0.23996 (14)	0.5369 (2)	0.73793 (6)	0.0247 (3)	
H3A	0.1922	0.6286	0.7607	0.030*	
C4	0.37662 (14)	0.57143 (19)	0.71688 (6)	0.0223 (3)	
H4A	0.4232	0.6876	0.7247	0.027*	
C5	0.44366 (13)	0.43305 (18)	0.68430 (6)	0.0188 (2)	
C6	0.18551 (15)	0.05222 (19)	0.67584 (7)	0.0246 (3)	
C7	0.59368 (14)	0.46266 (18)	0.66116 (6)	0.0210 (3)	
C8	0.99015 (14)	0.53539 (19)	0.59952 (6)	0.0213 (3)	
C9	1.12726 (14)	0.5468 (2)	0.57398 (6)	0.0225 (3)	
H9A	1.1815	0.6589	0.5776	0.027*	
C10	1.18187 (14)	0.3971 (2)	0.54415 (6)	0.0236 (3)	
C11	1.09980 (15)	0.2309 (2)	0.53908 (7)	0.0262 (3)	
H11A	1.1361	0.1252	0.5189	0.031*	
C12	0.96820 (15)	0.2250 (2)	0.56356 (7)	0.0260 (3)	
H12A	0.9122	0.1143	0.5601	0.031*	
C13	1.32713 (15)	0.4067 (2)	0.51713 (7)	0.0304 (3)	
H13A	1.3607	0.5359	0.5172	0.046*	
H13B	1.3184	0.3600	0.4745	0.046*	
H13C	1.3971	0.3305	0.5420	0.046*	

### Atomic displacement parameters (Å^2^)

**Table:**

	*U*^11^	*U*^22^	*U*^33^	*U*^12^	*U*^13^	*U*^23^
O1	0.0254 (5)	0.0214 (5)	0.0463 (6)	−0.0024 (4)	0.0069 (4)	−0.0036 (4)
O2	0.0241 (5)	0.0321 (6)	0.0420 (6)	−0.0091 (4)	0.0083 (4)	0.0018 (5)
O3	0.0212 (5)	0.0213 (5)	0.0446 (6)	−0.0042 (4)	0.0074 (4)	−0.0023 (4)
O4	0.0197 (4)	0.0216 (5)	0.0390 (6)	0.0005 (4)	0.0097 (4)	−0.0001 (4)
O1W	0.0315 (5)	0.0227 (5)	0.0377 (6)	0.0012 (4)	0.0043 (4)	−0.0029 (4)
O2W	0.0253 (10)	0.0408 (12)	0.0271 (10)	0.0025 (9)	0.0032 (8)	−0.0036 (9)
N1	0.0168 (5)	0.0204 (5)	0.0208 (5)	0.0003 (4)	0.0027 (4)	0.0018 (4)
N2	0.0180 (5)	0.0242 (6)	0.0251 (5)	−0.0012 (4)	0.0040 (4)	0.0003 (4)
N3	0.0209 (6)	0.0264 (6)	0.0345 (7)	−0.0019 (5)	0.0076 (5)	−0.0054 (5)
C1	0.0181 (6)	0.0217 (6)	0.0201 (6)	−0.0010 (5)	0.0022 (4)	0.0027 (5)
C2	0.0188 (6)	0.0295 (7)	0.0219 (6)	0.0004 (5)	0.0046 (5)	0.0015 (5)
C3	0.0220 (6)	0.0287 (7)	0.0236 (6)	0.0040 (5)	0.0039 (5)	−0.0045 (5)
C4	0.0202 (6)	0.0216 (6)	0.0249 (6)	0.0000 (5)	0.0011 (5)	−0.0032 (5)
C5	0.0163 (5)	0.0207 (6)	0.0196 (6)	0.0008 (5)	0.0026 (4)	0.0010 (5)
C6	0.0235 (6)	0.0224 (6)	0.0280 (7)	−0.0027 (5)	0.0030 (5)	0.0041 (5)
C7	0.0177 (6)	0.0212 (6)	0.0242 (6)	−0.0007 (5)	0.0022 (5)	0.0015 (5)
C8	0.0182 (6)	0.0239 (6)	0.0219 (6)	0.0003 (5)	0.0019 (5)	0.0011 (5)
C9	0.0175 (6)	0.0263 (7)	0.0239 (6)	−0.0020 (5)	0.0025 (5)	0.0022 (5)
C10	0.0169 (6)	0.0325 (7)	0.0217 (6)	0.0024 (5)	0.0032 (5)	0.0034 (5)
C11	0.0248 (6)	0.0275 (7)	0.0266 (7)	0.0038 (5)	0.0038 (5)	−0.0028 (5)
C12	0.0248 (6)	0.0244 (7)	0.0288 (7)	−0.0012 (5)	0.0019 (5)	−0.0018 (5)
C13	0.0207 (6)	0.0409 (8)	0.0304 (7)	0.0028 (6)	0.0082 (5)	0.0023 (6)

### Geometric parameters (Å, °)

**Table:**

O1—C6	1.3204 (17)	C2—C3	1.384 (2)
O1—H1O	0.93 (2)	C2—H2A	0.9500
O2—C6	1.2179 (17)	C3—C4	1.3937 (18)
O3—C7	1.2528 (16)	C3—H3A	0.9500
O4—C7	1.2632 (16)	C4—C5	1.3904 (18)
O1W—H1WA	0.8500	C4—H4A	0.9500
O1W—H1WB	0.8500	C5—C7	1.5206 (17)
O2W—H2WB	0.8500	C8—C9	1.4188 (17)
O2W—H2WA	0.8501	C9—C10	1.3701 (19)
N1—C1	1.3375 (16)	C9—H9A	0.9500
N1—C5	1.3379 (17)	C10—C11	1.416 (2)
N2—C8	1.3494 (17)	C10—C13	1.5019 (18)
N2—C12	1.3583 (18)	C11—C12	1.3598 (19)
N2—H2N	0.96 (2)	C11—H11A	0.9500
N3—C8	1.3320 (18)	C12—H12A	0.9500
N3—H3NB	0.978 (19)	C13—H13A	0.9800
N3—H3NA	0.90 (2)	C13—H13B	0.9800
C1—C2	1.3872 (18)	C13—H13C	0.9800
C1—C6	1.4966 (19)		
			
C6—O1—H1O	114.2 (15)	O2—C6—C1	122.14 (13)
H1WA—O1W—H1WB	113.8	O1—C6—C1	117.35 (12)
H2WB—O2W—H2WA	102.8	O3—C7—O4	125.49 (12)
C1—N1—C5	117.44 (11)	O3—C7—C5	117.50 (12)
C8—N2—C12	122.13 (12)	O4—C7—C5	117.01 (11)
C8—N2—H2N	119.4 (12)	N3—C8—N2	118.49 (12)
C12—N2—H2N	118.5 (12)	N3—C8—C9	123.32 (13)
C8—N3—H3NB	118.6 (11)	N2—C8—C9	118.18 (12)
C8—N3—H3NA	119.0 (13)	C10—C9—C8	120.37 (13)
H3NB—N3—H3NA	121.7 (17)	C10—C9—H9A	119.8
N1—C1—C2	124.10 (12)	C8—C9—H9A	119.8
N1—C1—C6	115.21 (12)	C9—C10—C11	119.22 (12)
C2—C1—C6	120.69 (12)	C9—C10—C13	121.01 (13)
C3—C2—C1	118.01 (12)	C11—C10—C13	119.77 (13)
C3—C2—H2A	121.0	C12—C11—C10	118.97 (13)
C1—C2—H2A	121.0	C12—C11—H11A	120.5
C2—C3—C4	118.79 (12)	C10—C11—H11A	120.5
C2—C3—H3A	120.6	N2—C12—C11	121.12 (13)
C4—C3—H3A	120.6	N2—C12—H12A	119.4
C5—C4—C3	118.91 (13)	C11—C12—H12A	119.4
C5—C4—H4A	120.5	C10—C13—H13A	109.5
C3—C4—H4A	120.5	C10—C13—H13B	109.5
N1—C5—C4	122.74 (11)	H13A—C13—H13B	109.5
N1—C5—C7	116.28 (11)	C10—C13—H13C	109.5
C4—C5—C7	120.97 (12)	H13A—C13—H13C	109.5
O2—C6—O1	120.49 (13)	H13B—C13—H13C	109.5
			
C5—N1—C1—C2	−1.24 (19)	N1—C5—C7—O3	168.20 (12)
C5—N1—C1—C6	178.42 (11)	C4—C5—C7—O3	−12.68 (19)
N1—C1—C2—C3	0.6 (2)	N1—C5—C7—O4	−12.13 (17)
C6—C1—C2—C3	−179.05 (12)	C4—C5—C7—O4	166.98 (12)
C1—C2—C3—C4	0.4 (2)	C12—N2—C8—N3	−179.79 (13)
C2—C3—C4—C5	−0.6 (2)	C12—N2—C8—C9	0.82 (19)
C1—N1—C5—C4	0.94 (18)	N3—C8—C9—C10	179.92 (13)
C1—N1—C5—C7	−179.97 (11)	N2—C8—C9—C10	−0.72 (19)
C3—C4—C5—N1	0.0 (2)	C8—C9—C10—C11	0.0 (2)
C3—C4—C5—C7	−179.08 (12)	C8—C9—C10—C13	−179.71 (13)
N1—C1—C6—O2	−176.83 (13)	C9—C10—C11—C12	0.5 (2)
C2—C1—C6—O2	2.8 (2)	C13—C10—C11—C12	−179.69 (13)
N1—C1—C6—O1	1.73 (18)	C8—N2—C12—C11	−0.2 (2)
C2—C1—C6—O1	−178.60 (12)	C10—C11—C12—N2	−0.5 (2)

### Hydrogen-bond geometry (Å, °)

**Table:**

*D*—H···*A*	*D*—H	H···*A*	*D*···*A*	*D*—H···*A*
O1W—H1WA···O4^i^	0.85	2.01	2.846 (2)	169
O1W—H1WA···N1^i^	0.85	2.50	2.935 (2)	112
O1W—H1WB···O3	0.85	1.97	2.813 (2)	169
O2W—H2WB···O1W^ii^	0.85	2.11	2.944 (2)	167
O2W—H2WA···O1W	0.85	2.07	2.919 (2)	175
O1—H1O···O1W^iii^	0.93 (3)	1.78 (3)	2.661 (2)	155 (2)
O1—H1O···N1	0.93 (3)	2.20 (2)	2.673 (2)	110 (2)
N2—H2N···O4	0.96 (2)	1.74 (2)	2.700 (2)	174 (2)
N3—H3NB···O3	0.98 (2)	1.86 (2)	2.829 (2)	172 (2)
N3—H3NA···O2^iv^	0.90 (2)	2.09 (2)	2.955 (2)	160 (2)
C2—H2A···O2^v^	0.95	2.47	3.158 (2)	129
C9—H9A···O1^iv^	0.95	2.56	3.399 (2)	147
C11—H11A···O2W^vi^	0.95	2.55	3.405 (2)	149
Cg(1)_N2/C8–C12_—···.Cg(1)^vi^	.	.	3.4690 (8)	.
Cg(1)—···.Cg(2)_N1/C1–C5_^vii^	.	.	3.6932 (8)	.
C7—O3···Cg(2)^viii^	.	3.5023 (12)	.	124.86 (9)

Symmetry codes: (i) *x*, *y*+1, *z*; (ii) −*x*+1, −*y*+2, −*z*+1; (iii) *x*, *y*−1, *z*; (iv) *x*+1, *y*+1, *z*; (v) −*x*, *y*+1/2, −*z*+3/2; (vi) −*x*+2, −*y*+1, −*z*+1; (vii) *x*+1, *y*, *z*; (viii) −*x*+1, *y*+1/2, −*z*+3/2.
